# GATA2^−/−^ human ESCs undergo attenuated endothelial to hematopoietic transition and thereafter granulocyte commitment

**DOI:** 10.1186/s13619-015-0018-7

**Published:** 2015-08-05

**Authors:** Ke Huang, Juan Du, Ning Ma, Jiajun Liu, Pengfei Wu, Xiaoya Dong, Minghui Meng, Wenqian Wang, Xin Chen, Xi Shi, Qianyu Chen, Zhongzhou Yang, Shubin Chen, Jian Zhang, Yuhang Li, Wei Li, Yi Zheng, Jinglei Cai, Peng Li, Xiaofang Sun, Jinyong Wang, Duanqing Pei, Guangjin Pan

**Affiliations:** 1 CAS Key Laboratory of Regenerative Biology, South China Institute for Stem Cell Biology and Regenerative Medicine, Guangzhou Institutes of Biomedicine and Health, Chinese Academy of Sciences, Guangzhou, 510530 China; 2 Guangdong Provincial Key Laboratory of Stem Cell and Regenerative Medicine, South China Institute for Stem Cell Biology and Regenerative Medicine, Guangzhou Institutes of Biomedicine and Health, Chinese Academy of Sciences, Guangzhou, 510530 China; 3 Department of Hematology, Sun Yat-sen University, Guangzhou, 510630 China; 4 South China University of Technology, Guangzhou, 510641 China; 5 Key Laboratory for Major Obstetric Diseases of Guangdong Province, Guangzhou, China; 6 Key Laboratory of Reproduction and Genetics of Guangdong Higher Education Institutes, Guangzhou, China

**Keywords:** hESCs, GATA2, EHT, HPC, Granulocyte, Notch signaling

## Abstract

**Background:**

Hematopoiesis is a progressive process collectively controlled by an elaborate network of transcription factors (TFs). Among these TFs, GATA2 has been implicated to be critical for regulating multiple steps of hematopoiesis in mouse models. However, whether similar function of GATA2 is conserved in human hematopoiesis, especially during early embryonic development stage, is largely unknown.

**Results:**

To examine the role of GATA2 in human background, we generated homozygous *GATA2* knockout human embryonic stem cells (*GATA2* ^*−/−*^ hESCs) and analyzed their blood differentiation potential. Our results demonstrated that *GATA2* ^*−/−*^ hESCs displayed attenuated generation of CD34^+^CD43^+^ hematopoietic progenitor cells (HPCs), due to the impairment of endothelial to hematopoietic transition (EHT). Interestingly, *GATA2* ^*−/−*^ hESCs retained the potential to generate erythroblasts and macrophages, but never granulocytes. We further identified that SPI1 downregulation was partially responsible for the defects of *GATA2* ^*−/−*^ hESCs in generation of CD34^+^CD43^+^ HPCs and granulocytes. Furthermore, we found that *GATA2* ^*−/−*^ hESCs restored the granulocyte potential in the presence of Notch signaling.

**Conclusion:**

Our findings revealed the essential roles of GATA2 in EHT and granulocyte development through regulating *SPI1*, and uncovered a role of Notch signaling in granulocyte generation during hematopoiesis modeled by human ESCs.

**Electronic supplementary material:**

The online version of this article (doi:10.1186/s13619-015-0018-7) contains supplementary material, which is available to authorized users.

## Background

Hematopoiesis is a complex process that involves multiple developmental processes, such as cellular proliferation, differentiation, and survival. This process is accurately controlled by the coordination of a set of transcription factors and diverse signaling pathways [1–5]. GATA2 belongs to the transcriptional regulatory GATA protein family and is broadly expressed in hematopoietic cells, particularly in hematopoietic progenitors [6, 7]. The essential function of GATA2 in genesis, differentiation, and even trans-differentiation of hematopoietic stem/or progenitor cells (HSCs or HPCs) has been extensively examined [8, 9]. A GATA2-deficient mouse exhibited severe anemia and died at early stage of gestation due to a reduced number of primitive erythroid cells and HPCs [10], highlighting the essential role of GATA2 in early hematopoiesis. Furthermore, GATA2 is also crucial in maintaining the proliferation and normal function of adult HSCs or HPCs [7, 11–13]. Recently, de Pater et al. demonstrated that GATA2-deficient hemogenic endothelium (HE) failed to generate long-term repopulating HSCs due to the impairment of endothelial to hematopoietic transition (EHT) [14]. Mechanistically, GATA2 might regulate HPCs through direct activation of other critical factors. For instance, Pimanda et al. described that Gata2, Fli1, and Scl/Tal1 formed a regulatory circuit to regulate early hematopoietic development in the mouse model [1].

Besides HSCs or HPCs, GATA2 also regulates hematopoietic lineage specification. For example, overexpression of GATA2 in primary erythroid progenitor cells promoted megakaryocyte differentiation while inhibiting erythrocyte differentiation [15]. Adult bone marrow from GATA2 heterozygous mice (*GATA2* ^+/−^) exhibited reduced function of granulocyte-macrophage progenitors (GMPs) [16]. The diverse roles of GATA2 in different hematopoietic lineages indicate that its function is largely cell context-dependent [17, 18]. Given that most of the data available now on how GATA2 regulates hematopoiesis are obtained from the murine system, its roles in human background remain elusive and require further investigation.

Human embryonic stem cells (hESCs) are capable of hematopoietic differentiation in vitro and thus could serve as a valuable model for investigating early human hematopoiesis. They could efficiently differentiate into HPCs as well as different hematopoietic lineages, through either co-culturing with stromal cells or embryoid body (EB) formation in the presence of specific cytokines [19, 20]. The hESC-derived HPCs exhibited typical phenotype of blood progenitors, including expressing surface markers as well as forming different blood lineage colonies (colony-forming cells, CFCs). Moreover, most studies to date support that the in vitro blood differentiation of hESCs was a controlled sequential process starting from the early embryonic mesoderm, via HE and HPCs to mature blood cells, recapitulating hematopoietic development in vivo [21, 22]. Therefore, it could serve as a good system to examine the role of GATA2 during early human hematopoiesis.

In this report, through gene targeting, we generated *GATA2* ^*−/−*^ human ESCs and analyzed their hematopoietic differentiation potential. Through examining surface markers that were previously identified in hESC-derived HPCs (CD34^+^CD43^+^) [23, 24], we found that *GATA2* ^*−/−*^ hESCs generated much less HPCs both in the OP9 co-culturing system and a stromal-free medium that could drive blood differentiation. However, *GATA2* ^*−/−*^ hESCs retained the potential to produce the major subtype blood lineages, such as erythroblasts and macrophages. In contrast, we observed a complete defect of *GATA2* ^*−/−*^ hESCs in generating granulocytes in OP9-driven blood differentiation. Mechanistically, we identified that the granulocyte defect was partially due to the downregulation of *SPI1*, a critical transcription factor known for myeloid and lymphocyte development in the mouse model. Enforced expression of *SPI1* rescued the production of granulocytes of *GATA2* ^*−/−*^ hESCs in co-culturing with OP9. Interestingly, *GATA2* ^*−/−*^ hESCs restored the potential when co-culturing with OP9 expressing DL1, the Notch signaling ligand. Thus, our findings revealed the critical roles of GATA2 in EHT and granulocyte development in human-modeled hematopoiesis.

## Results

### Generation of *GATA2*^*−/−*^ human ESCs

**Fig. 1:**
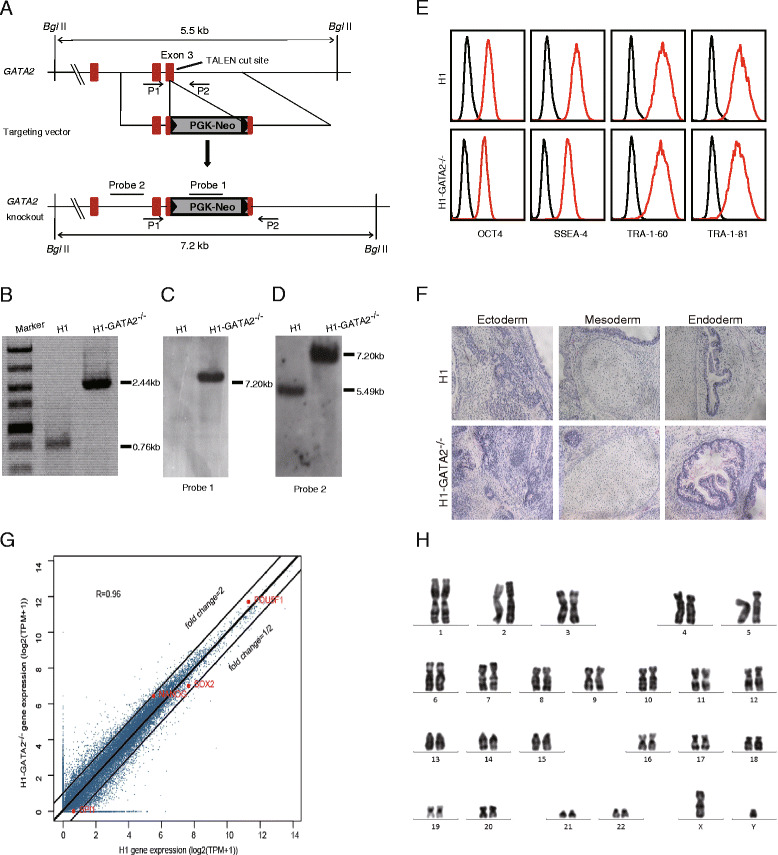
Generation of *GATA2* ^*−/−*^ human ESCs. **a** Schematic overview of gene targeting strategy. For *GATA2* knockout, a PGK promoter-neomycin cassette replaces exon 3 of the *GATA2* locus. *Bgl*II sites, TALEN sites, primers (*P1*, *P2*), and probes (*probe 1*, *probe 2*) used for genomic PCR and Southern blot are indicated. **b** Genomic PCR for *GATA2* targeting. PCR product by indicated primers is 0.76 kb from WT H1 cells and 2.44 kb from H1-*GATA2* ^**−/−**^. **c**, **d** Southern blot analysis of *GATA2* targeted H1 cells. The genomic DNAs were digested by *Bgl*II and detected by indicated probes. One single band detected by probe 1 indicates that there was no random integration of drug cassette. **e** FACS analysis of the expression of OCT4, SSEA-4, TRA-1-60, and TRA-1-81 in H1 or H1-*GATA2* ^**−/−**^ cells. In these and other flow cytometry diagrams, the *black line* stands for the control and the *red line* for the experimental plot unless otherwise indicated. **f** Teratoma formation of H1 or H1-*GATA2* ^**−/−**^ cells. **g** Paired Pearson correlation analysis of global gene expression between WT and *GATA2* ^*−/−*^ H1 cells. *R* Pearson correlation coefficient, *TPM* transcripts per million. **h** Karyotype analysis of the H1-*GATA2* ^*−/−*^ cell line

Gene targeting in hESCs could be significantly improved with the aid of specifically designed nucleases such as zinc finger nucleases (ZFNs) or transcription activator-like effector nucleases (TALENs) [25–27]. In order to generate GATA2 null human ES cells, we firstly constructed a targeting vector in which a PGK-driven neomycin resistance cassette inserted into exon 3 of the *GATA2* gene (Fig. 1a). To facilitate gene targeting, we designed a pair of TALENs specifically recognized and cut the targeting site of the human *GATA2* gene. Based on a reporter assay for analyzing the efficiency of TALENs [28], we showed that the TALENs were highly specific and efficient (Additional file 1: Figure S1A-C). Then, we introduced the linearized targeting vector together with the TALENs into H1 hESCs through electroporation and selected the positive clones using neomycin. After selection, drug-resistant colonies were manually picked for further expansion and screening by genomic PCR and Southern blot. Eventually, we successfully expanded an H1 hESC clone with homozygous disruption of *GATA2* alleles (*GATA2* ^*−/−*^) (Fig. 1b–d). The disruption could result in the inability of these cells to generate functional GATA2 protein even though an aberrant mRNA might be generated (Additional file 1: Figure S1D). *GATA2* ^*−/−*^ H1 hESCs maintained under undifferentiated condition kept a normal karyotype and exhibited similar characteristics to the wild-type (WT) counterpart regarding surface marker expression, global gene expression profile, as well as teratoma formation (Fig. 1e–h). Furthermore, *GATA2* ^*−/−*^ H1 could form typical EBs with upregulation of markers for three early embryonic germ layers during random differentiation (Additional file 1: Figure S2). In summary, *GATA2* disruption did not generate obvious alterations in hESCs under conditions for hESC self-renewal and random differentiation.

### *GATA2*^*−/−*^ hESCs generate reduced HPCs due to EHT defect

**Fig. 2:**
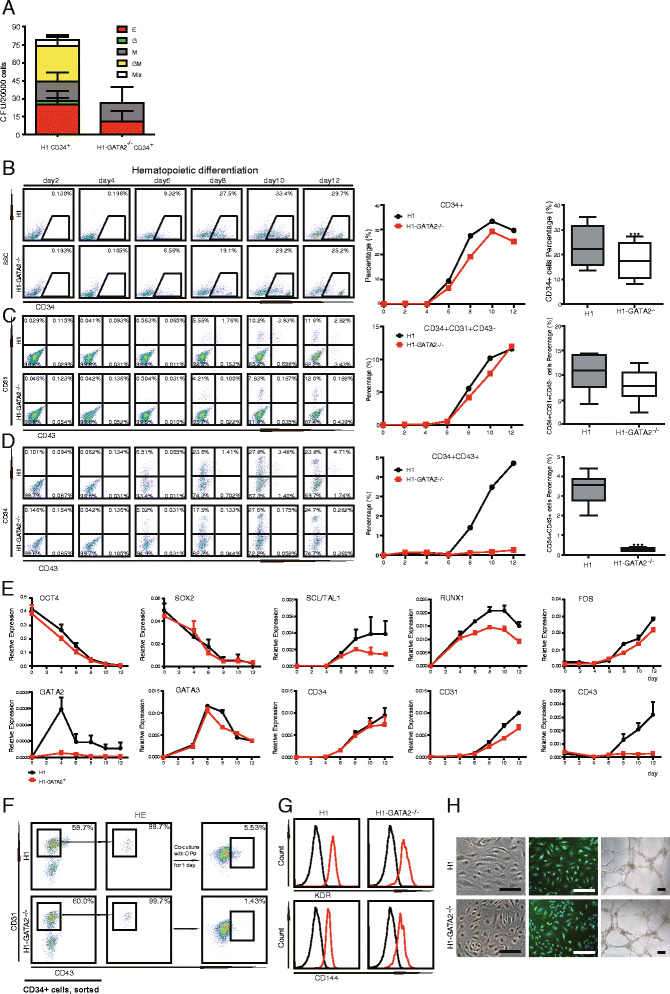
Hematopoietic differentiation of the H1-*GATA2* ^*−/−*^ ES cell line. **a** CFUs of H1 or H1-*GATA2* ^*−/−*^ derived CD34^+^ cells. H1 or H1-*GATA2* ^*−/−*^ cells were co-cultured with OP9 cells for 9 days. The CD34^+^ HPCs were isolated by FACS for CFU generation. *Error bars* represent mean + SEM of the mean of samples from nine independent experiments. **b-d** Time course analysis of blood differentiation of H1 and H1-*GATA2* ^*−/−*^ cells upon co-culturing with OP9. The expression of surface markers CD34, CD43, and CD31 on H1 or H1-*GATA2* ^*−/−*^ cells co-cultured with OP9 for the indicated time was analyzed by FACS (*left* and *middle panels*). The *right panels* are box plots from ten independent experiments on the percentage of indicated populations on day 8 of OP9 co-culturing. *Asterisks* indicate statistical significance determined by *t* test: ***p < 0.001. **e** Time course analysis of the expression of indicated genes of H1 and H1-*GATA2* ^*−/−*^ cells upon co-culturing with OP9. The gene expression was analyzed by qRT-PCR by using GAPDH as an internal reference. **f** HPCs with CD34^+^CD31^+^CD43^+^ were developed from CD34^+^CD31^+^CD43^−^ HEs. CD34^+^CD31^+^CD43^−^ populations were sorted out at day 8 of differentiation and replated on OP9 for one additional day and analyzed by FACS. **g** FACS analysis of CD114 and KDR on HEs from H1 or H1-*GATA2* ^*−/−*^. **h** H1 or H1-*GATA2* ^*−/−*^ derived HEs produced endothelial cells. *Left*: Morphology of endothelial cells; *middle*: immunostaining of CD31 on endothelial cells; *right*: capillary structure formation by endothelial cells

Since GATA2 has been known to be a master regulator for hematopoiesis, we sought to analyze the hematopoietic potential of *GATA2* ^*−/−*^ hESCs. In a stromal-free defined condition that could drive blood differentiation, we showed that in contrast to WT hESCs, *GATA2* ^*−/−*^ hESCs generated a few HPCs (CD34^+^CD43^+^) and blood colony-forming units (CFUs) (Additional file 1: Figure S3). However, when co-culturing with OP9 stromal cells, *GATA2* ^*−/−*^ hESCs exhibited CFC potential (Fig. 2a). This data is consistent with previous findings in vivo in the mouse model that mouse ES cells lacking Gata2 could generate certain blood lineages, such as erythrocytes [10]. We confirmed that the *GATA2* ^*−/−*^ hESCs failed to express full-length *GATA2* mRNAs during the whole process of blood differentiation driven by OP9 co-culture (Fig. 2e and Additional file 1: Figure S1D). Then, we attempted to analyze this process in detail. Upon OP9 co-culture, the HPCs with CFU potential were believed to develop through the EHT process from HEs, the endothelial cells with hematopoietic potential [21, 22, 29–35]. Through further analyzing the surface markers at different differentiation stages during OP9 co-culture, we showed that *GATA2* ^*−/−*^ hESCs exhibited little difference in the generation of CD34^+^CD31^+^ HEs compared with WT hESCs, but significant reduction in the production of CD34^+^CD43^+^ HPCs (Fig. 2b–d). These data indicated that GATA2 was critical for EHT to generate HPCs but not essential for HE determination. Consistently, the transcription factors (TFs) critical for HE determination such as RUNX1 and SCL/TAL1 [30] were successfully activated in *GATA2* ^*−/−*^ hESCs, albeit a little lower than in WT hESCs (Fig. 2e, Additional file 1: Figure S4).

To further analyze the function of HEs from *GATA2* ^*−/−*^ hESCs, we sorted out the CD34^+^CD31^+^CD43^−^ HEs at day 8 of OP9 co-culture. Upon replating them onto OP9 cells for further hematopoietic differentiation, HEs from *GATA2* ^*−/−*^ hESCs produced much less CD43^+^ HPCs compared with WT hESCs (Fig. 2f). Nevertheless, the HEs derived from both WT and *GATA2* ^*−/−*^ hESCs expressed typical endothelium markers such as KDR and CD144 (VE-Cadherin) (Fig. 2g). In addition, to explore their endothelial potential, we re-cultured the sorted HEs in endothelial growth medium onto a Matrigel-coated plate. In this condition, *GATA2* ^*−/−*^ HEs underwent further endothelial differentiation that displayed a typical endothelial phenotype and formed vascular tubes, which are comparable to WT HEs (Fig. 2h). In general, our data showed that GATA2 null hESCs could develop into functional HE lineages, but undergo deficient EHT to produce HPCs.

### Characterization of hematopoietic potential of GATA2 null HPCs

**Fig. 3:**
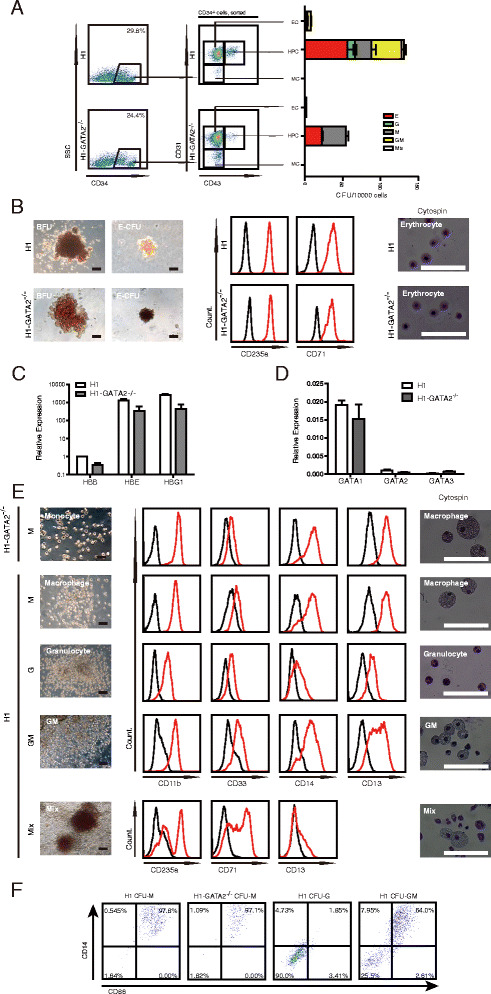
Characterization of subtype blood lineages from H1 or H1-*GATA2* ^*−/−*^ derived HPCs. **a** CFU potential cells from H1 or H1-*GATA2* ^*−/−*^ were restricted within CD34^+^CD43^+^ subpopulations. *EC* endothelial cells, *MC* mesenchymal cells. **b** Characterization of erythrocytes from H1 or H1-*GATA2* ^*−/−*^. From *left* to *right*: phase-contrast photographs of BFU and CFU-E, FACS analysis of CD235a and CD71a expression on H1 and H1-*GATA2* ^*−/−*^ derived erythrocytes, and cytospin of H1 and H1-*GATA2* ^*−/−*^ derived erythrocytes. **c** Globin analysis of erythrocytes by RT-qPCR. The results showed the mean + SEM of one single experiment with three replicates, representative of three independent experiments. **d** Analysis of expression of GATA1, GATA2, and GATA3 in H1 or H1-*GATA2* ^*−/−*^ derived erythrocytes. The results showed the mean + SEM of one single experiment with three replicates, representative of three independent experiments. **e** Characterization of myeloid cells from H1 or H1-*GATA2* ^*−/−*^. *Left*: morphologies of indicated CFU colonies; *middle*: FACS analysis of indicated markers; *right*: cytospin photographs of indicated colonies. **f** FACS analysis of CD86 and CD14 expression in H1 and H1-*GATA2* ^*−/−*^ derived myeloid CFU. *E* erythrocyte, *G* granulocyte, *M* macrophage, *GM* G and M, *Mix* G, E, and M

As shown in Fig. 2a, although we could easily detect the major colony-forming cell (CFC) types including erythroid (CFC-E or BFU) and myeloid (granulocytes and macrophages, CFU-Mix) from WT hESCs upon co-culturing with OP9, we only observed erythroid and macrophage CFCs from *GATA2* ^*−/−*^ CD34^+^ cells. We never detected granulocytes (CFU-G) from *GATA2* ^*−/−*^ CD34^+^ cells. Consistent with previous findings, we showed that the potential cells were restricted within the population of CD34^+^CD43^+^ (Fig. 3a) [23]. These data indicated that GATA2 might particularly target and regulate granulocyte specification in human ESC-based hematopoiesis.

We then further performed comprehensive characterization of all myeloid lineages generated from *GATA2* ^*−/−*^ hESCs/OP9 co-culture. Regarding in vivo embryonic hematopoiesis, two distinct hematopoietic programs, the primitive hematopoiesis and definitive hematopoiesis, have been demonstrated to produce different subtype blood lineages [36]. The primitive hematopoiesis occurred early and mainly produced primitive erythroblasts and macrophages, while the definitive hematopoiesis was associated with definitive erythroid expressing adult beta-globin and pan-myeloid precursors [24]. Both distinct programs have been detected during in vitro hESCs/OP9 co-culturing, but it remains largely challenging to precisely define and separate these two stages [37]. In our system, we could easily detect the erythroid precursors from *GATA2* ^*−/−*^ hESCs/OP9 co-culture, characterized by forming E-CFUs or even BFUs (Fig. 3c) with expression of CD235a and CD71, the well-established markers for in vitro-generated erythroid cells [33] (Fig. 3b). Furthermore, we could detect the expression of both adult globin (HBB) and embryonic globins (HBE and HBG) in erythrocytes from *GATA2* ^*−/−*^ hESCs/OP9 (Fig. 3c). Consistently, GATA1, the critical factor known for erythroid specification, was successfully activated and detected in *GATA2* ^*−/−*^ erythrocytes (Fig. 3d).

In contrast, myeloid lineages from *GATA2* ^*−/−*^ hESCs exhibited significant morphological difference to those from WT hESCs (Fig. 3e). While multiple myeloid lineages such as macrophage (M-CFCs), granulocyte (G-CFCs), and pan-myeloid CFCs (CFU-Mix) were observed from WT hESCs, we only observed mononuclear cells from *GATA2* ^*−/−*^ hESCs, which displayed macrophage morphology under a microscope (Fig. 3e). To further confirm the identity of mononuclear cells from *GATA2* ^*−/−*^ HPCs, we performed fluorescence-activated cell sorting (FACS) analysis of these cells with specific surface markers. For these surface markers, macrophages highly express both CD11b and CD14 while granulocytes express high CD11b but low CD14; thus, they could be used to discriminate these two lineages as reported by Rafii et al. [33]. Consistently, we showed that *GATA2* ^*−/−*^ hESC-derived myeloid cells displayed high expression of both CD11b and CD14, demonstrating that these cells were macrophages, but not granulocytes (Fig. 3e). In another literature, Choi et al. reported that CD14 was expressed in both granulocytic and monocytic cells generated in vitro [38]. Thus, we also examined an additional surface marker to separate these two populations, for example, CD86, which is highly expressed in monocytes/macrophages, but lowly expressed in granulocytes [39]. We found that the *GATA2* ^*−/−*^ hESC-derived myeloid cells exclusively expressed high level of CD86 (Fig. 3f), further demonstrating that *GATA2* ^*−/−*^ hESCs produced macrophages (M-CFCs), not granulocytes (G-CFCs). Further, Giemsa staining confirmed the morphology for macrophage, but not granulocyte for these monocytes (Fig. 3e). Overall, these data revealed an essential role of GATA2 in regulating granulocyte generation during human ESC-modeled hematopoiesis.

### SPI1 was responsible for the HPC and granulocyte defects of *GATA2*^*−/−*^ hESCs

**Fig. 4:**
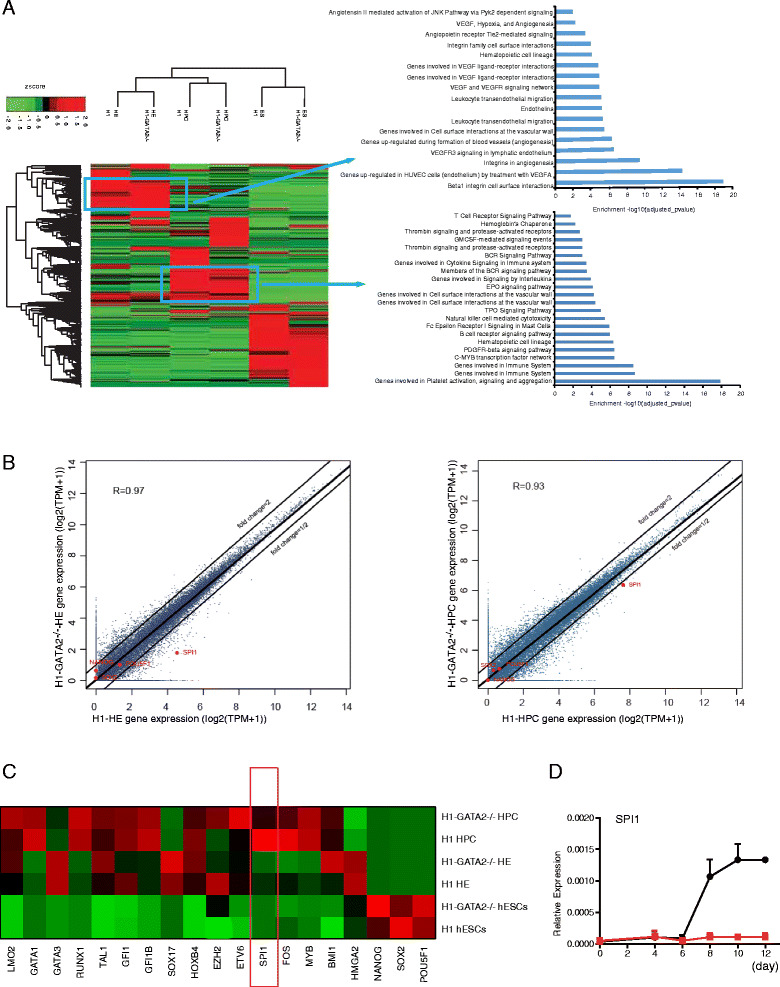
Global gene expression analyses of H1 or H1-*GATA2* ^*−/−*^ derived HPC and HE. **a** Hierarchical clustering analysis of RNA-Seq data for indicated samples. Genes with TPMs above 1 and at least threefold change compared with each of any other sample were selected for the analysis. Gene expression values were normalized by *Z* score and clustered using Clustergram software. The enriched biological functions of the indicated gene group were analyzed by Gene Ontology (GO). HPC: CD34^+^CD43^+^ cells sorted at day 9 of co-culture; HE: CD34^+^CD31^+^CD43^−^ cells sorted at day 8 of co-culture. **b** Paired Pearson correlation analysis of H1 and H1-*GATA2* ^*−/−*^ derived HEs (*left*) or HPCs (*right*). *R* Pearson correlation coefficient, *TPM* transcripts per million. Selected genes (*red*) are highlighted. **c** Heat map of selected genes based on TPM value of indicated samples. **d** Time course analysis of SPI1 expression during the OP9 co-culture by qRT-PCR. *Error bars* represent SEM of the mean of one single experiment with three replicates, representative of three independent experiments

To probe the underlying mechanisms of EHT and granulocyte defects of *GATA2* ^*−/−*^ hESCs, we performed RNA-Seq analysis at different hematopoietic differentiation stages. Through hierarchical cluster analysis of the whole genome expression data, we showed that the samples at the same differentiation stage were highly correlated, regardless of *GATA2* status (Fig. 4a). Genes upregulated in HEs and HPCs were enriched for the biological function in blood and vessel development, indicating that *GATA2* deficiency did not abolish the general blood lineage specification. Indeed, further comparison of HEs or HPCs from *GATA2* ^*−/−*^ hESCs with their counterparts from WT hESCs revealed much less difference on the global expression profile (Fig. 4b). In order to figure out the reason for *GATA2* ^*−/−*^ HPC failure in the generation of granulocytes, we examined numbers of genes that were known as critical hematopoietic regulators, particularly those downregulated in H1-*GATA2* ^*−/−*^ derived HE and/or HPC (Fig. 4c). Even though most of these regulators exhibited less difference between *GATA2* ^*−/−*^ and WT cells, we did find some differentially expressed genes, for example, *SPI1*. SPI1 is a transcription factor that is highly expressed in myeloid and known to be critical for proper development and function of these lineages [40]. Further, through qRT-PCR analysis, we confirmed that *GATA2* ^*−/−*^ hESCs failed to activate *SPI1* upon co-culture with OP9 (Fig. 4c). These data suggested that *SPI1* might be the downstream target for GATA2 to regulate EHT and granulocyte development.

### Forced expression of SPI1 rescued HPC and granulocyte defects in *GATA2*^*−/−*^ hESCs

**Fig. 5:**
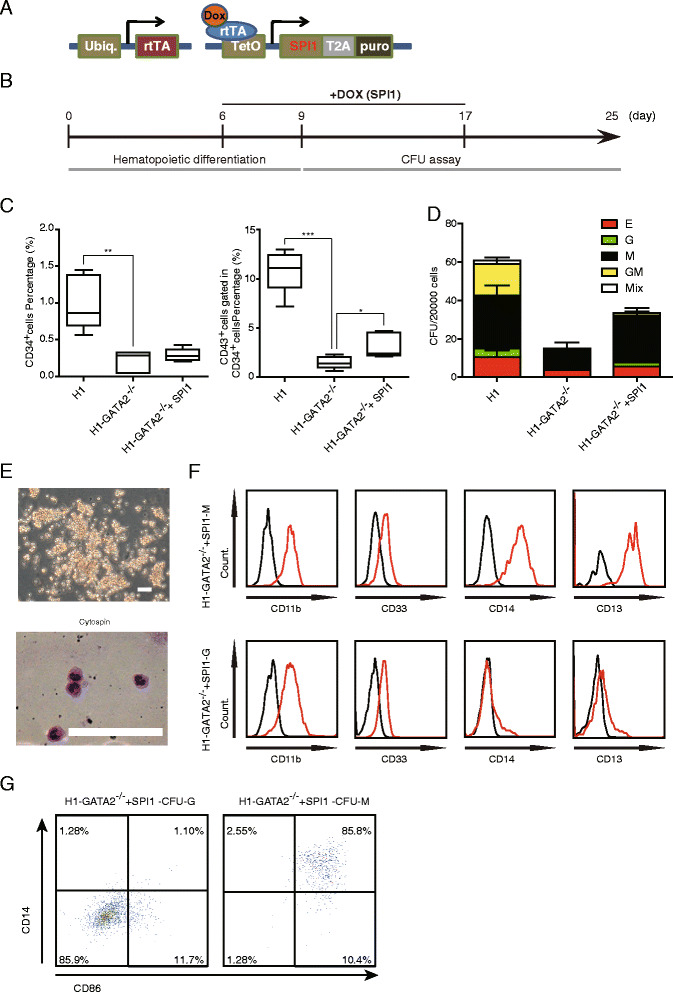
Forced expression of SPI1 in H1-*GATA2* ^*−/−*^ restores the generation of granulocytes upon OP9 co-culture. **a**, **b** Diagram of the strategy of SPI1 rescue experiments. SPI1 linked with a puromycin resistance gene by T2A sequence was controlled by a Dox-inducible promoter in lentiviral-based vectors for Dox-inducible expression of SPI1. The expression of SPI1 was not induced during later CFU assay. **c** Effects of enforced expression of SPI1 on generation of in CD34^+^ (*left*) and CD34^+^CD43^+^ (*right*) HPCs in H1-*GATA2* ^*−/−*^. Results are presented as mean + SEM of five independent experiments and normalized to H1 group. The data on CD34^+^ cells generation (*left*) were set as 1 for comparison. The data from five independent experiments were shown as box plot. *Asterisks* indicate statistical significance determined by *t* test: *p < 0.05, **p < 0.01 and ***p < 0.001. **d** Enforced expression of SPI1 in H1-*GATA2* ^*−/−*^ regenerate G-CFUs. The *error bars* indicate mean + SEM of three independent experiments. **e** Morphology of CFU-G regenerated by SPI1 expression; *bottom*: cytospin of CFU-G. **f**, **g** FACS analysis of indicated markers in SPI1 regenerated CFU-M and CFU-G from H1-GATA2^−/−^

A mouse embryo with homozygous mutation of *SPI1* died at later gestation stage with a complete loss of B cells, T cells, and macrophages, demonstrating the essential role of *SPI1* in the proper development of such lineages [41], and SPI1 is one of the four genes used by Sandler et al. to induce hematopoietic MPPs from HUVECs [42]. However, the role of SPI1 in granulocyte development has been conflicting and not yet well documented. Therefore, we sought to examine whether activation of SPI1 could rescue the granulocyte defect of *GATA2* ^*−/−*^ hESCs. To this end, we constructed an inducible vector expressing SPI1 in a lentiviral backbone [43] and introduced it into *GATA2* ^*−/−*^ hESCs (Fig. 5a), which could be expressed with Dox addition (Additional file 1: Figure S5). Because *SPI1* only started to express at day 6 of differentiation in WT hESCs/OP9 co-culturing (Fig. 4d), we just induced the expression of *SPI1* starting at day 6 of differentiation and withdrew the induction during subsequent CFC assay (Fig. 5b). Upon forced induction of SPI1, we showed that CD34^+^CD43^+^ HPCs were significantly restored in *GATA2* ^*−/−*^ hESCs/OP9 co-culture (Fig. 5c). The granulocyte potential cells (G-CFC) did re-appear in *GATA2* ^*−/−*^ hESCs/OP9 with forced expression of *SPI1* (Fig. 5d). However, consistent with the previous finding that SPI1 could antagonize with GATA1 to suppress erythropoiesis [44], we observed a lower level of E-CFCs in *GATA2* ^*−/−*^ hESCs/OP9 with forced expression of SPI1 (Fig. 5d). Nonetheless, the granulocytes re-established by SPI1 exhibited typical morphology and surface marker expression compared with those from WT hESCs (Fig. 5e–g). In summary, we demonstrated that *SPI1* was the target of *GATA2* to ensure normal development of HPCs and granulocytes in human ESC-modeled hematopoiesis, thus highlighting a precise and specific role of *GATA2* and *SPI1* in the regulation of EHT and granulocyte generation.

### *GATA2*^*−/−*^ hESCs partially restored the granulocyte potential in the presence of Notch signaling

**Fig. 6:**
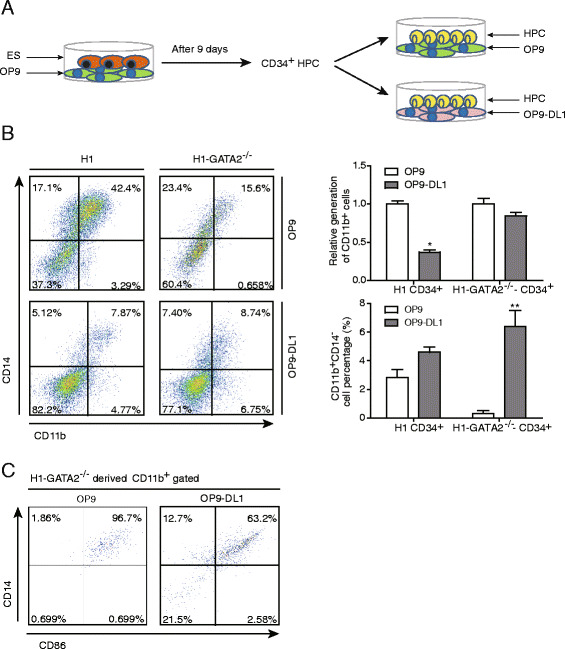
H1-*GATA2* ^*−/−*^ cells restored the potential of granulocyte on OP9-DL1. **a** Diagram of the strategy of the experiments. H1 or H1-*GATA2* ^*−/−*^ ES cells were co-cultured with OP9 for 9 days, then the CD34^+^ HPCs were harvested and seeded onto OP9 or OP9-DL1 cells for myeloid differentiation. **b** CD11b and CD14 expression at day 12 of OP9/OP9-DL1-mediated myeloid differentiation were analyzed by FACS. Percentage of total CD11b^+^ myeloid cells or CD11b^+^CD14^−^ granulocytes were shown at the *left*. The *right* bar charts represent the statistic results of relative generation of CD11b^+^ cells of indicated test (*up*) and the generation of CD11b^+^CD14^−^ cells (*down*). The data of H1 and H1-*GATA2* ^*−/−*^ from the OP9 co-culture for CD11b^+^ cell generation were set as 1 for comparison. Results indicate mean + SEM of three independent experiments. *Asterisks* represent statistical significance determined by *t* test: *p < 0.05 and **p < 0.01. **c** FACS analysis of CD86 and CD14 expression in day 12 of OP9/OP9-DL1-mediated myeloid differentiation

It has been shown that Notch signaling could inhibit myelopoiesis on normal stem cells and uncommitted hematopoietic progenitors. This inhibition largely depended on the normal function of GATA2 and could be rescued after GATA2 knockout [45]. However, it remained elusive whether granulocyte commitment could be restored after GATA2 knockout. Therefore, we seeded WT or *GATA2* ^*−/−*^ hESC-derived CD34^+^ HPCs directly onto wild-type OP9 or OP9 expressing DL1 (OP9-DL1), the Notch signaling ligand for further myeloid differentiation with addition of cytokines [46] (Fig. 6a). As shown in Fig. 6b, the total number of CD11b^+^ myeloid cells generated from WT hESCs was significantly reduced upon co-culturing with OP9-DL1 compared with OP9, indicating that Notch signaling indeed inhibits myelopoiesis in human cells. However, the Notch-mediated inhibition of myelopoiesis was not obvious on *GATA2* ^*−/*^ hESC-derived CD34^+^ HPCs (Fig. 6b, upper panel), demonstrating that GATA2 was the critical downstream factor for Notch signaling to inhibit myelopoiesis. Furthermore, we showed that the CD11b^+^CD14^−^ granulocytes from *GATA2* ^*−/−*^ hESCs were significantly restored in the presence of Notch signaling (Fig. 6b, lower panel). Additional markers, such as CD86, also confirmed the generation of granulocytes of *GATA2* ^*−/−*^ hESCs on OP9-DL1 (Fig. 6c). These findings demonstrate that the granulocyte potential of *GATA2* ^*−/−*^ hESCs could be restored in the presence of Notch signaling.

## Discussion

Currently, studies on hematopoiesis by gene knockout approach have been limited to model organisms, such as zebra fish and mice, while studies on the genetic determinants of human hematopoiesis have been confined to overexpression and knockdown in human pluripotent stem cells. In this report, we proved that human ESCs combined with gene knockout could be utilized to investigate early human hematopoiesis by demonstrating hematopoietic defects generated through deletion of a key hematopoietic transcription factor GATA2 from human ES cell line by TALEN.

The GATA family contains a series of factors that are evolutionally conserved and essential for proper development in mammals. Among them, *GATA1*, *GATA2*, and *GATA3* express and function predominantly in hematopoietic lineage cells [13, 47]. These factors exhibited spatial and temporal expression patterns among different hematopoietic lineages. For example, GATA2 expression displayed broad distribution but with a prominently high level in early hematopoietic progenitors [12]. The function of GATA factors have been extensively examined in the mouse model through various in vivo experimental approaches. It becomes more and more clear that GATA2 is required for the genesis and/or function of HSCs. However, the function of GATA factors in human hematopoiesis has not been clearly elucidated due to the limitation of embryonic materials. Here, taking the in vitro hematopoietic differentiation of human ESCs as a model, we were able to analyze the function of GATA2 in human background. Aided by TALENs to enhance genome editing, we generated human ESCs with homozygous mutation on *GATA2* (*GATA2* ^*−/−*^ hESCs). *GATA2* ^*−/−*^ hESCs behaved similarly to their wild-type counterparts.

In contrast, regarding hematopoietic differentiation, we observed that *GATA2* ^*−/−*^ hESCs generated much less HPCs marked by CD34^+^CD43^+^ based on the OP9 co-culture system, but retained the potential to differentiate into major types of hematopoietic lineages, such as erythrocyte and macrophage. Surprisingly, we failed to observed granulocyte differentiation from *GATA2* ^*−/−*^ hESCs. This is very different from previous reports. In the mouse model, Tsai and de Pater et al. reported that Gata2^−/−^ mouse ESCs are capable of producing multipotential CFCs including granulocytes albeit in a much smaller number compared to wild-type ESCs [10, 14], and Gao et al. documented the abolished CFC potential of Gata2^−/−^ AGM cells. Our findings indicated that the function of GATA2 would be very crucial for granulocyte development in human ESC-modeled hematopoiesis (Additional file 1: Figure S5). Meanwhile, it is worthy to note that the HPCs from *GATA2* ^*−/−*^ hESCs failed to generate any blood CFCs in the stromal-free system (Additional file 1: Figure S3), indicating that OP9 might provide additional factors or proper niche to allow CFC formation for *GATA2* ^*−/−*^ HPCs. In addition, the presence of Notch signaling in OP9 stromal cells restored the granulocyte potential of *GATA2* ^*−/−*^ ESCs. This finding is consistent with a previous report [45] and suggests that the effect of Notch signaling in GMP cells requires GATA2 and GATA2 is the downstream of Notch signaling to inhibit HPC commitment to myeloid lineage, and further highlights the important role of the interaction between the extracellular environment and intracellular gene regulation in blood cell development.

GATA2 was also found to play a role in vascular integrity. Mammoto et al. reported that knockdown of *GATA2* in endothelial cells impaired vascular formation of human endothelial cells both in vitro and in vivo [48]. Also, Kazenwadel et al. reported that GATA2 knockdown in primary lymphatic endothelial cells abolished the lymphatic endothelial cell marker expression of *FOXC2*, *PROX1*, *ITGA9*, *VEGFR3*, and *ANGPT2* [49]. However, Tsai et al. found that *GATA2* ^*−/−*^ embryos showed apparently normal endothelial cells, vitelline vasculature, and heart on E9.5 [10]. Consistent with the report of Tsai et al., we found that the endothelium and vascular formation potential of *GATA2* ^*−/−*^ hESCs were normal as demonstrated by capillary structure formation and we observed no general defects in the lymphatic endothelial cell marker expression after *GATA2* mutation. This may be due to a complementary mechanism which compensates for the deletion of *GATA2*.

At the molecular level, several factors have been reported to be direct downstream targets of GATA2 at the HSC stage. For example, SCL/Tal1, a critical factor that controls survival of HPCs at early hematopoiesis, seems to be directly activated and maintained by GATA2 in mouse models [1]. Runx1, another important factor for normal hematopoiesis, was also directly targeted and maintained by Gata2 in mouse HPCs [50]. However, these factors as well as some well-known hematopoietic factors were successfully activated in the absence of GATA2 upon hematopoietic differentiation of human ESCs in our system (Fig. 4c), which might explain why most blood lineages could be generated. However, *SPI1*, a critical gene, which has been reported to be involved in myeloid development, was significantly downregulated in *GATA2* ^*−/−*^ HE and HPCs. Although the partial rescue effect of *SPI1* may be due to the overdose effect of forced *SPI1* expression, it may also imply that other candidate targets of GATA2 contribute to EHT and/or granulocyte development yet not carefully examined currently.

*GATA2* ^*−/−*^ hESCs failed to express *SPI1* upon in vitro hematopoiesis through co-culturing with OP9, and *GATA2* has been reported to directly target and activate the *SPI1* locus in mouse HPCs through two conserved regions [51]. GATA2 has been reported to be involved in EHT [8, 14, 52], whereas the role of *SPI1* in EHT has not been well documented. Until very recently, Adam et al. reported that *Spi1* was upregulated during the EHT process [53], and in another milestone study, Sandler et al. reported reprogramming of human endothelial cells to transplantable hematopoietic progenitor cells by *FOSB*, *GFI1*, *RUNX1*, and *SPI1* induction [42]. These studies combined with our report proved that *SPI1* would potentially serve as an important regulator in EHT. Nevertheless, it is still worthy to note that *Gata2* might be with specific functions in SPI1 regulation in different cell types. For instance, Gata2 could bind to the Cebpa promoter, blocking Spi1 and Runx1 binding, and so prevents *Cebpa* gene activation for the maintenance of cellular identity of mast cells [54].

Previous studies on the role of *SPI1* in granulopoiesis have been conflictive in some degree. *Spi1* has been proved to be crucial for HSC maintenance and myeloid differentiation [55], and *Spi1* mutant embryos exhibited multilineage defects including the impairment of granulocytes [56], while other studies reported that *Spi1* ^−/−^ granulocyte-monocyte progenitors (GMP) can differentiate into granulocytic precursors but with further maturation impairment [57] and elimination of *Spi1* in GMP in adult mice showed disturbed hematopoiesis with excess granulocyte production [58]. These studies indicated specific roles of *SPI1* at different stages of granulocyte development. They implied that dysfunction of *SPI1* impairs HSC generation and its commitment to downstream lineages including granulocyte, while *SPI1* is not essential for the differentiation of GMP to granulocyte. In our study, we showed that *SPI1* could rescue the HPC generation of *GATA2* ^*−/−*^ hESCs and the restored HPC is with granulocyte potential. These results confirmed the role of *SPI1* in HPC generation and its differentiation to granulocyte in mice and further highlight its conserved role in regulating myeloid development during hematopoiesis (Additional file 1: Figure S6).

## Conclusion

In conclusion, we reported the first study of human hematopoiesis through gene knockout and illustrated the roles of GATA2 and SPI1 in EHT and granulocyte generation in early human hematopoiesis. Particularly, we revealed the impact of interaction between Notch signaling and GATA2 on granulocyte development.

## Methods

### Targeting strategy

GATA2 knockout TALENs were designed as described [25, 26], and their sequences and targeting site were illustrated in Additional file 1: Figure S1. For donor construction, left and right homology arms were cloned from genomic DNA of the H1 cell line. A loxP-flanked PGK-neomycin cassette was further inserted between two homology arms in the vector pUC57. The vector is linearized by *Eco*RI before targeting. For targeting, 1.5 × 106 H1 cells were electroporated with 1 μg of donor DNA and 2.5 μg of each TALEN plasmid. Then, the cells were seeded on a Matrigel-coated six-well plate in the presence of Y-27632 (10 μM, Sigma). After 2 or 3 days, positive clones were selected by G418 (100 μg/ml, Sigma). Further verifications were carried out by genomic PCR and Southern blot. All primers referred are listed in Additional file 1: Table S1.

### Hematopoietic colony-forming assays

Hematopoietic colony-forming assays were performed in 35-mm culture dishes (Stem Cell Technologies, Inc.) using 1 ml per dish of MethoCult™ H4435 enriched medium (Stem Cell Technologies, Inc.) mixed with cells of a certain number according to the manufacturer’s instructions. Colonies were counted on days 14–16 and picked individually, washed in FACS buffer, and spun onto slides with a cytospin apparatus (TXD-3). The cells were then fixed and processed with Wright-Giemsa staining.

### FACS analysis and cell sorting

For GFP fluorescence analysis, cells were trypsinized and suspended in FACS buffer (PBS with 2 % FBS (ExCell)) directly for detection by C6 (BD Accuri). For cell surface antigen analysis, cells were stained with antibody cocktail in FACS buffer at 4 °C for 15 to 30 min after trypsinization. Specifically, to analyze day 9 subsets from co-culture, cells were stained with CD43-FITC, CD31-PE, CD34-PerCP-Cy5.5, and TRA-1-85-APC. To analyze and sort HEs in day 8 and HPCs in day 9 of co-culture, CD34^+^ cells were primarily isolated by MACS (Miltenyi Biotech) and subsequently sorted using a FACSAria cell sorter (BD Biosciences) with cells stained by CD43-FITC, CD31-PE, CD34-PerCP-Cy5.5, and TRA-1-85-APC. To analyze endothelial markers expressed on HEs, cultured HEs were stained by CD144-PE and KDR-PE, respectively. To analyze colony-forming unit (CFU) surface makers, CFU-E were stained with CD235a-PE and CD71a APC; CFU-G/M/GM or myeloid cells derived from HPCs co-cultured with OP9/OP9-DL1 were stained with CD11b-FITC, CD33-PE, CD14-PerCP-Cy5.5, CD13-APC or CD11b-FITC, CD86-PE, CD14-PerCP-Cy5.5; CFU-Mix cells were stained with CD235a-PE, CD13-PerCP-Cy5.5, and CD71a-APC. To analyze the surface maker expressed on pluripotent stem cells, they were stained with no-conjugated primary SSEA-4, TRA-1-60, and TRA-1-81 antibody, respectively, and further stained with species-specific secondary antibodies conjugated to Alexa Fluor® 448 (Invitrogen). For intracellular antigen OCT4, PAX6, and NESTIN analysis, cell fixation and permeabilization were performed before antibody incubation and then cells were stained with primary and secondary antibodies as SSEA4. Particularly, for cell sorting, after staining with antibodies, cells were further stained with DAPI excluding dead cells; the purity of sorted fractions was more than 97 % as tested by FACS. All antibodies used for FACS analysis are listed in Additional file 1: Table S2.

Additional methods are listed in the Additional file 1.

### Accession numbers

The RNA-Seq data are available in the Gene Expression Omnibus database [accession number: GSE69797].

## Additional file

Additional file 1: **Supplemental information includes seven supplemental figures, supplemental figure legends, two supplemental tables, and the extended methods.** (PDF 1270 kb)

## Abbreviations

CFCs: colony-forming cells
CFU: colony-forming unit
E: erythrocyte
EHT: endothelial to hematopoietic transition
G: granulocyte
GM: granulocyte and macrophage
GMP: granulocyte-macrophage progenitor
HE: hemogenic endothelium
hESCs: human embryonic stem cells
HPCs: hematopoietic progenitor cells
HSCs: hematopoietic stem cells
M: macrophage
