# Supplementary material for: GATA2−/− human ESCs undergo attenuated endothelial to hematopoietic transition and thereafter granulocyte commitment
Source: Cell Regen. 2015 Aug 5;4:4. doi: 10.1186/s13619-015-0018-7 (PMC4526303; doi:10.1186/s13619-015-0018-7)
Supplement: Supplementary file 1 — Supplemental information includes seven supplemental figures, supplemental figure legends, two supplemental tables, and the extended methods. (PDF 1270 kb) [file 13619_2015_18_MOESM1_ESM.pdf]

Supplementary Figure 1

A

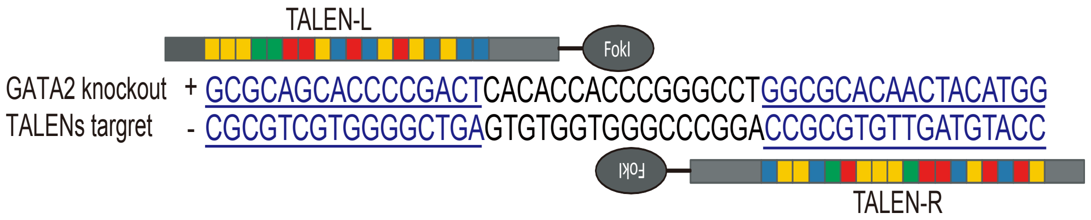

Wildtype	5' GCGCAGCACCCCGACTCACACCACCCGGGCCTGGCGCACAACTACATGG 3'
Mut1: Mutation	5' ACACAACACCCCAACTCACACCACCCGGGCCTAACACACAACTACATAA 3'
Mut2: Similar sequence 1	5' GACTCACACCACCCGG 3'
Mut3: Similar sequence 2	5' CACCCGGGCCTTGAGCACAACTA 3'
Mut4: Similar sequence 3	5' GCAGCACCCGGACACGCACCACCCGGGCCT 3'
Mut5: Left sequence	5' GCGCAGCACCCCGACTCACACCACCCGGGCCT------------------------------ 3'
Mut6: Right sequence	5' --------------------------- CACACCACCCGGGCCTGGCGCACAACTACATGG 3'

B

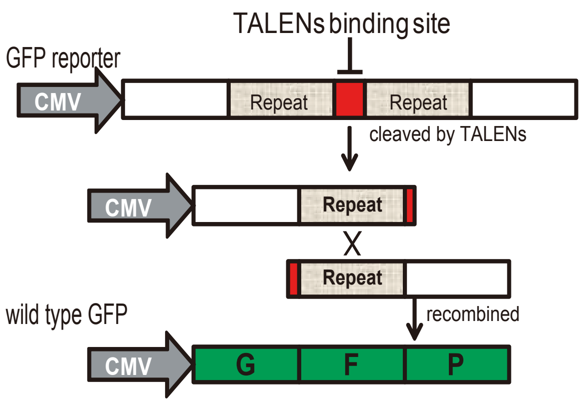

C

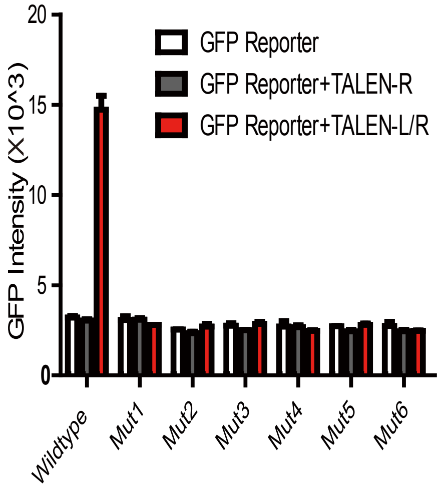

D

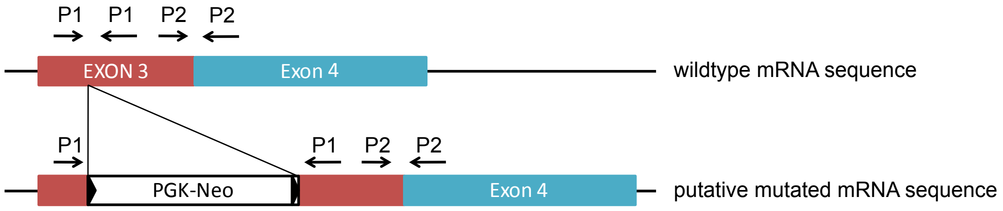

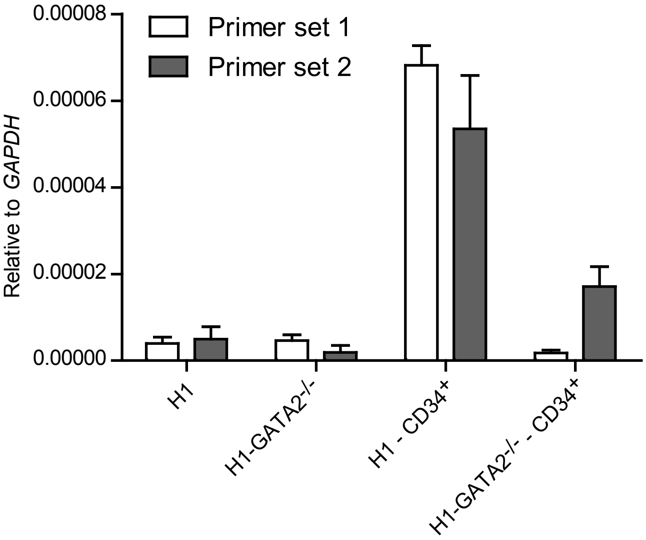

Supplemental Figure 2

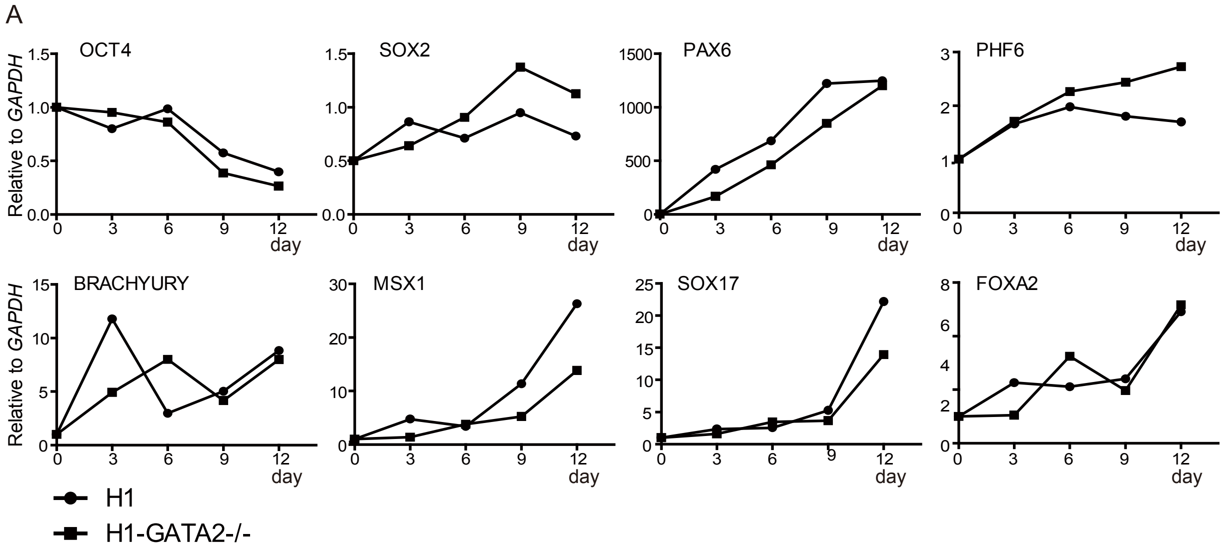

Supplemental Figure 3

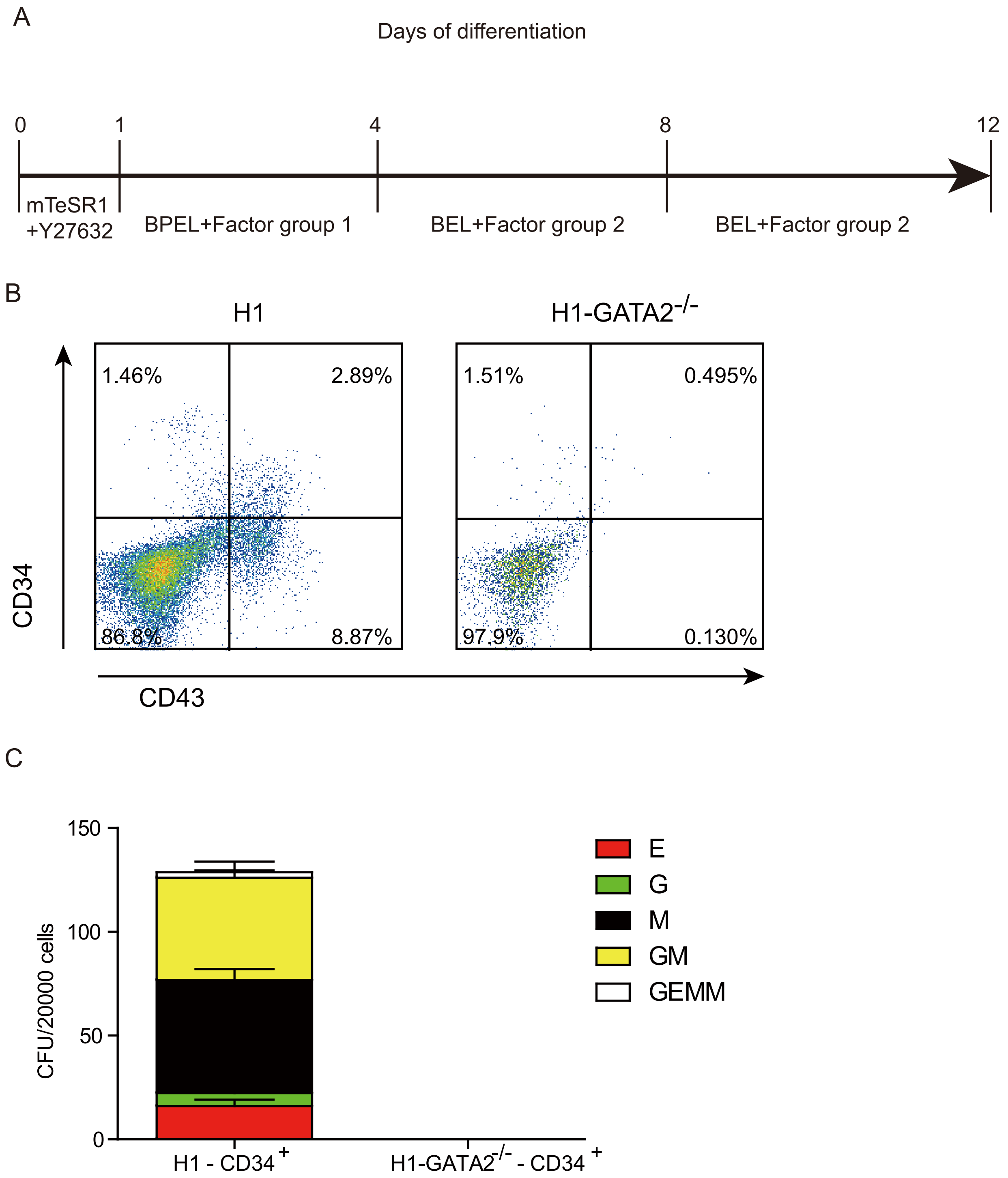

Supplemental Figure 4

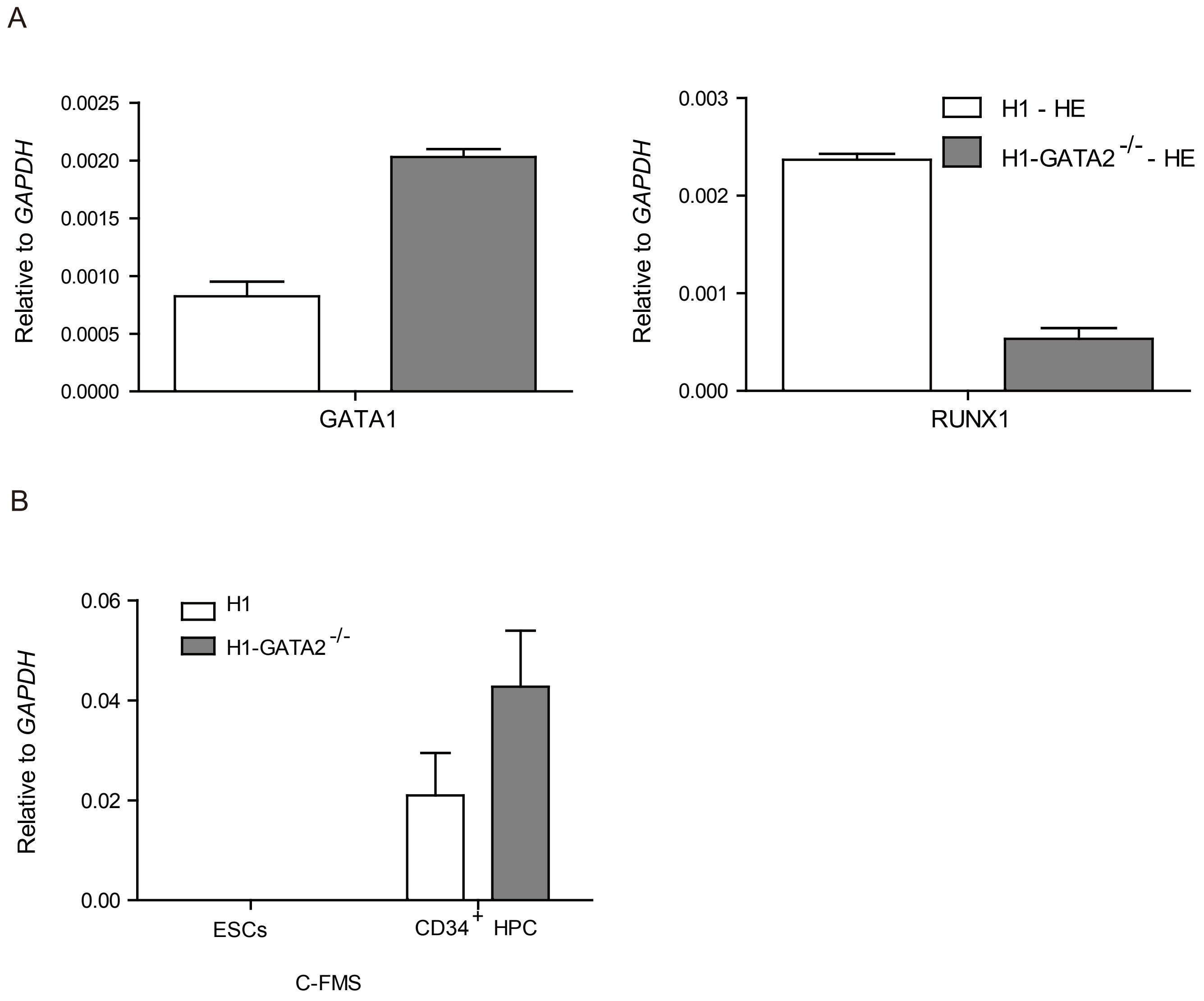

Supplemental Figure 5

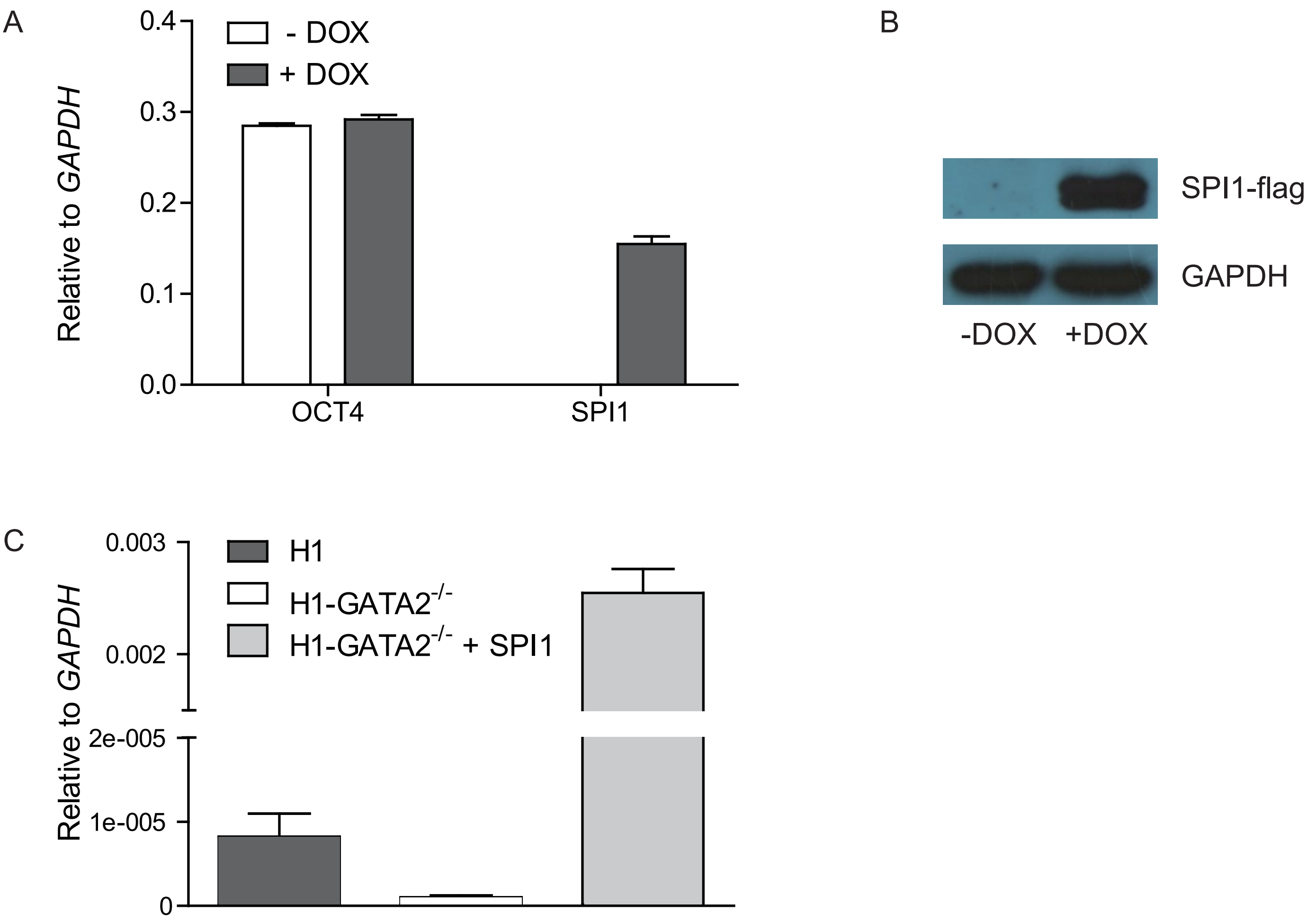

Supplemental Figure 6

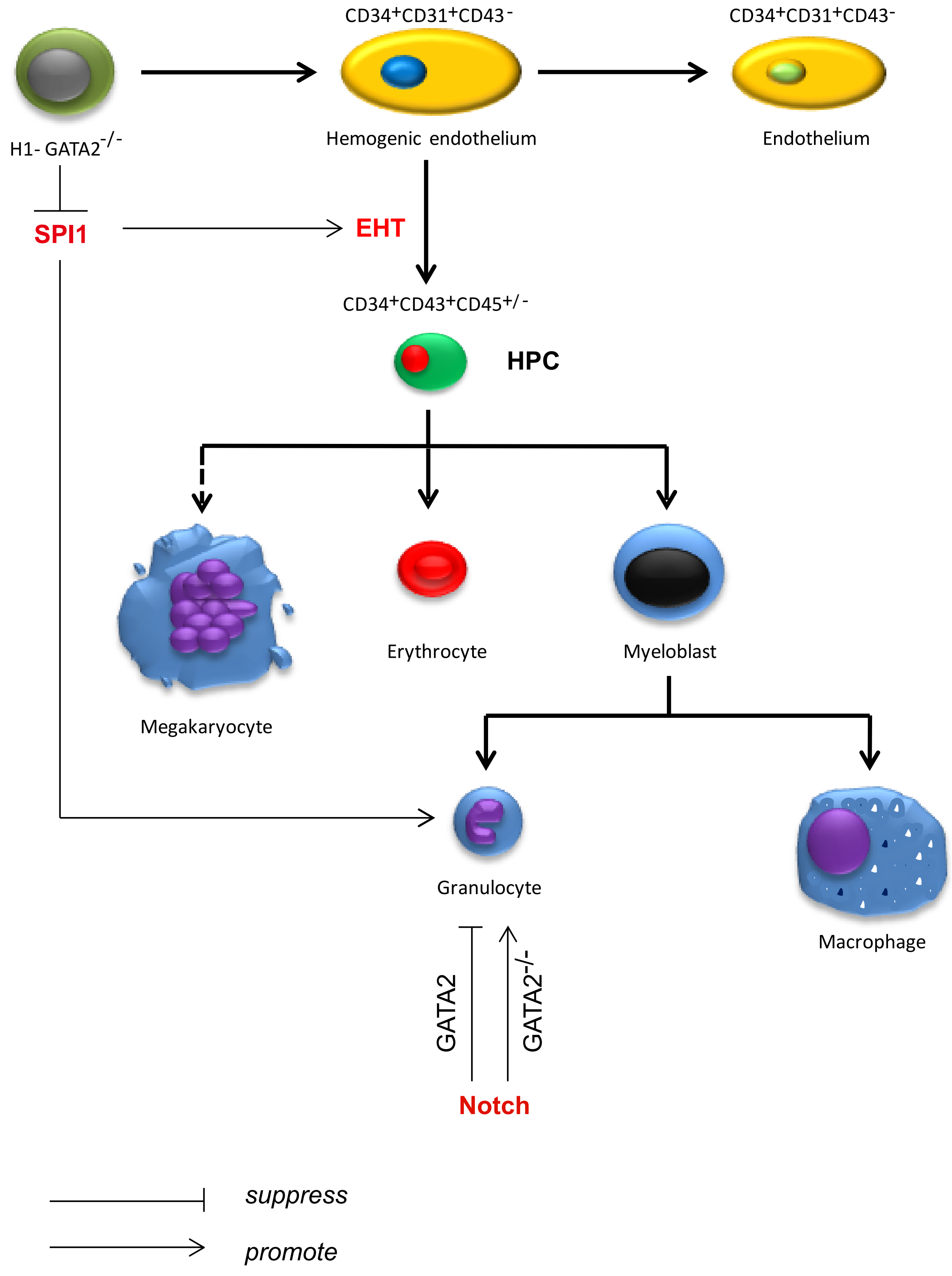

Supplemental Figure legends

Figure S1 TALENs for human *GATA2* editing

(A) *Top*: the recognition sequence of TALENs in Exon 3 of GATA2; *bottom*: different mutation types in the recognition sequence of GATA2 knockout TALENs for GFP reporter assay. **(B)** Schematic design for the GFP reporter assay. **(C)** Specificity of designed GATA2 knockout TALENs. 293T cells were harvested and GFP fluorescence was tested by FACS at 48 hours after cells were transfected with GFP reporter with different TALEN recognition sequences and the TALENs. **(D)** *Top*: design of qPCR primers for GATA2 knockout verification; *bottom*: qPCR verification results of GATA2 expression in indicated cells. The sense and antisense primer of Primer set 1 locates between the cut sides of TALENs; the sense and antisense primer of Primer set 2 locates on Exon3 (on the downstream of cut site in detail) and Exon 4 respectively. GATA2 is expressed in both H1 and H1-*GATA2*$^{-/-}$ derived CD34^{+} cells but not in hESCs. Primer set 1 cannot amplify the mutated sequence in normal RT-qPCR procedure due to the length of mutant sequence, thus could be used to identify the mutation of GATA2. Primer 1 was used to detect GATA2 expression in this and other figures unless otherwise indicated. P1: Primer set 1; P2: Primer set 2.

Figure S2 Analysis of EB based differentiation of H1 or H1-*GATA2*$^{-/-}$.

(A) Time course analysis of indicated genes in EB random differentiation of H1 and H1-*GATA2*$^{-/-}$ ES cells.

Figure S3 Hematopoietic differentiation of H1 and H1-*GATA2*$^{-/-}$ cell lines in an exclusively cytokines dependent EB system.

(A) Differentiation scheme used for cytokines dependent hematopoietic differentiation. EBs were generated during the first 24 hours of culture in mTeSR1 supplemented with Y27632 (2μM) and BMP4 (20 ng/ml) in 24-well-plate with 300,000 cells per well. Subsequently, EBs were collected and cultured in BPEL for three days in the presence of Factor group 1 (BMP4, ACTIVIN A, SCF and VEGF with a concentration of 20 ng/ml respectively). Then, EBs were cultured in BEL with Factor group 2 (BMP4, IL3, IL6, TPO (20 ng/ml) and FLT3-L, SCF (50 ng/ml)), and changed the medium every 4 days. At day 12 of differentiation, EBs were collected and digested for analysis. **(B)** Results indicated the FACS analysis of CD34^{+}CD43^{+} cells in EBs at day 12 of differentiation, representative of three independent experiments. C. CFU assay

of H1 and H1-*GATA2*$^{-/-}$ derived CD34^{+} cells in EB system. Results indicate mean + SEM of three independent experiments.

Figure S4 Key transcriptional factor expressed in HE and HPC

(A) qRT-PCR analysis of GATA1 (left) and RUNX1 (right) expression in both H1 and H1-*GATA2*$^{-/-}$ derived hemogenic endothelium (HE). **(B)** C-FMS expression level in both H1 and H1-*GATA2*$^{-/-}$ ES and derived CD34^{+} HPC.

Figure S5 Dox-inducible expression of SPI1

(A) qRT-PCR (*left*) and western blot (*right*) results of SPI1 expression in response to Dox addition of H1-*GATA2*$^{-/-}$ + *SPI1* cell line. (B) SPI1 expression in CD34^{+} cells generated from indicated cell lines.

Figure S6 Schematic representation of *GATA2*$^{-/-}$ impact on EHT and granulocyte development

GATA2 knockout hESCs give rise to normal endothelial cells and relative low level of HPCs, and could generate most of blood lineages except granulocytes. GATA2 knockout results in SPI1 downregulation; and SPI1 suppression further leads to HPC and granulocyte generation impairment as it is involved in both EHT and granulocyte development. Besides, notch singling blocks the granulocyte generation in condition of normal GATA2 function, but could rescue the granulocyte potential after GATA2 knockout.

Supplemental Table 1

Primer	Sequence 5' – 3'
GAPDH-F	TCCAAAATCAAGTGGGGCGAT
GAPDH -R	TTCTAGACGGCAGGTCAGGTC
OCT4-F	CCTCACTTCACTGCACTGTA
OCT4-R	CAGGTTTTCTTTCCCTAGCT
SOX2-F	CCCAGCAGACTTCACATGT
SOX2-R	CCTCCCATTTCCCTCGTTTT
NANOG-F	TGAACCTCAGCTACAAACAG
NANOG -R	TGGTGGTAGGAAGAGTAAAG
SOX17-F	CGCTTTCATGGTGTGGGCTAAGGACG
SOX17-R	TAGTTGGGGTGGTCCTGCATGTGCTG
PAX6-F	ACCCATTATCCAGATGTGTTTGCCCGAG
PAX6-R	ATGGTGAAGCTGGGCATAGGCGGCAG
MSX1-F	CGAGAGGACCCCGTGGATGCAGAG
MSX1-R	GGCGGCCATCTTCAGCTTCTCCAG
FOXA2-F	GGAGCAGCTACTATGCAGAGC
FOXA2-R	CGTGTTCATGCCGTTCATCC
BRACHYURY-F	TATGAGCCTCGAATCCACATAGT
BRACHYURY-R	CCTCGTTCTGATAAGCAGTCAC
PHF6-F	AGACAGCGCAAATGTGGCTT
PHF6-R	TCAGCTTCGTGCCTCTTTTAAT
SCL/TAL1-F	AGCCGGATGCCTTCCCTAT
SCL/TAL1-R	GGGACCATCAGTAATCTCCATCT
RUNX1-F	CTGCCCATCGCTTTCAAGGT
RUNX1-R	GCCGAGTAGTTTTCATCATTGCC
FOS-F	CCGGGGATAGCCTCTCTTACT
FOS-R	CCAGGTCCGTGCAGAAGTC
GATA1-F	CTGTCCCCAATAGTGCTTATGG
GATA1-R	GAATAGGCTGCTGAATTGAGGG
GATA2-F (Primer 1)	GCCGTGCTGAATGCGC
GATA2-R (Primer 1)	CCTGCGAGTCGAGGTGATTG
GATA2-F (Primer 2)	GCAACCCCTACTATGCCAACC
GATA2-R (Primer 2)	CAGTGGCGTCTTGGAGAAG
GATA3-F	GCCCCTCATTAAGCCCAAG
GATA3-R	TTGTGGTGGTCTGACAGTTCG
CD31-F	AACAGTGTTGACATGAAGAGCC

CD31-R	TGTAAAACAGCACGTCATCCTT
CD34-F	CTACAACACCTAGTACCCTTGGA
CD34-R	GGTGAACACTGTGCTGATTACA
CD43-F	GCTGGTGGTAAGCCCAGAC
CD43-R	GGCTCGCTAGTAGAGACCAAA
SPI1-F	GTGCCCTATGACACGGATCTA
SPI1-R	AGTCCCAGTAATGGTCGCTAT
HBB-R	CCGAGCACTTTCTTGCCATGA
HBG1-F	TCACAGAGGAGGACAAGGCTACTAT
HBG1-R	CCTATCCTTGAAAGCTCTGAATCAT
HBE-F	ATGGTGCATTTTACTGCTGAGG
HBE-R	GGGAGACGACAGGTTTCCAAA
P1	CGGGTGTGTGATTCTGGAGC
P2	ATGCCACTTGTCCTTCAGCTTG
Probe1-F	GAGGCGCTTTTCCCAAGGCAGTCT
Probe1-R	CGCATTGCATCAGCCATGATGGATA
Probe2-F	GGGAGGGAACGGTCTGGGTAGGTAA
Probe2-R	GCCTCTGAGAGTGAAGGAGTTCCGG

Supplemental Table 2

Name	Conjugation	Cat#	Vendor
CD43	FITC	560978	BD Pharmingen
CD31	PE	555446	BD Pharmingen
CD34	PerCP-Cy5.5	347203	BD Pharmingen
TRA-1-85	APC	FAB3195A	R&D
CD144	PE	560410	BD Pharmingen
KDR	PE	560494	BD Pharmingen
CD235A	PE	MHGLA04	Invitrogen
CD71a	APC	FAB2474A	R&D
CD3	V500	UCHT1	Bioscience
CD4	PerCP-Cy5.5	RPA-T4	Bioscience
CD8	FITC	555366	BD Pharmingen
Nkp46	PE	557991	BD Pharmingen
CD19	APC-Cy7	557791	Bioscience
TCR-αβ	APC	17-9986-41	Bioscience
TCR-γδ	PE	12-9959-41	Bioscience
CD19	APC	17-0198-42	Bioscience
CD10	FITC	11-0108-42	Bioscience
CD31	none	sc-1506	Santa Cruz Biotechnology
OCT4	none	sc-5279	Santa Cruz Biotechnology
SSEA-4	none	414000	Invitrogen
TRA-1-60	none	MAB4360	Millipore
TRA-1-81	none	MAB4381	Millipore
PAX6	Alaxa Fluor 488	561664	BD Pharmingen
NESTIN	PerCP-Cy5.5	561231	BD Pharmingen

Extended methods

1. GFP reporter assay

For GFP reporter construction, both wild type and mutated sequences (Figure S1A) were inserted into "TALENs binding site" respectively (Figure S1B). Then, the constructed GFP reporter plasmids and TALENs were co-delivered into 293T cells at 80% confluence through calcium phosphate transfection. For 12-well plates, 1.5 μg of each TALEN and 1μg of reporter plasmids were used per well. If the TALENs could recognize the sequence, then they will cut it and after the incision of the sequence, intramolecular recombination could be triggered and a functional GFP protein could be formed in the reporter. Then GFP fluorescence would be detected. After 48 hours of the plasmids transfection, cells were harvested and GFP fluorescence was analyzed by FACS.

2. PCR and southern blot verification

PCR was performed by KOD-Plus (Toyobo) according to the manufacturer's instructions and 50-100 ng of genomic DNA templates were used for all reactions. Primer P1 and P2 (in the left and right homology arm respectively) were used to amplify a 2.44-kb product as illustrated in Figure 1B). Southern blot was carried out following the instruction manuals of DIG High Prime DNA Labeling and Detection Starter Kit Ⅱ (Roche). Particularly, genomic DNA was digested by Bgl *II*, and probes about 1 kb in length are synthesized by PCR amplification. Probe 1 was used to identify whether random integration occurred in the genome, and Probe 2 was used to verify if targeting was occurred in cutting site as designed. All primers and probe referred are listed in Supplementary Table1.

3. Teratoma formation and analysis

Confluent cells in a 10-cm-dish were digested by EDTA and resuspended in 300μL F12 medium, then mixed with 600μL Matrigel and injected subcutaneously and intramuscularly into immunodeficiency mice. Eight weeks later, teratomas were harvested, fixed in 4% paraformaldehyde (PFA) and analyzed by hematoxylin/eosin (HE) staining.

4. Karyotype analysis.

Cells used for karyotype analysis were grown in 10-cm-dish. Demecolcine (Dahui Biotech) was added to culture dish to reach a final concentration of 50 μg/ml for 40 min. Then, cells were trypsinized, pelleted by centrifugation at

300*g* for 3 min, then resuspended in 8 ml of 0.075 M hypotonic KCl solution and incubated at 37 °C for 20 min. Fixative solution composed of 1/4 acetic acid and 3/4 methanol was added to a final volume of 10 ml, gently mixed and incubated at 37 °C for 10 min. After further centrifugation, supernatant was discarded and ice-cold fixative solution was added to a final volume of 10 ml. Fixed cells were spread on a dried cold slide by dropping followed by incubation at 75 °C for 3 hours. The resulting belts were treated with trypsin and colorant, and cells at metaphase states were captured with an Olympus BX51 microscope.

5. RNA-Seq

After the digestion of cultured ES cell clone or flow sorting of selected cell population, the target cells were pelleted and lysed with 200ul Trizol (Invitrogen). Total RNA was prepared with Direct-zol RNA MiniPrep kit (Zymo Research) following the manufacture’s protocol. The resulting RNA was then purified, fragmented, reverse transcribed, labeled and amplified to generate sequencing-ready cDNA library with TruSeq RNA Sample Prep Kit (Illumina). A size selection step was included to purify cDNA libraries to enrich for 250-300 bp fragments instead of AMPure XP beads purification. The DNA was recovered from each gel slice using QIAquick gel extraction kit (QIAGEN). The cDNA library concentration was determined with Qubit dsDNA HS Assay kit (Invitrogen). Additional sample concentrating step was included if the library concentration falls below required loading amount. The samples were run on MiSeq system with MiSeq Reagent Kits v2 (50 cycles) (Illumina).

Barcoded samples were de-multiplexed and the single end read length is 30~50bp. FastQC (v0.10.1) performed quality control on raw sequence data (http://www.bioinformatics.babraham.ac.uk/projects/fastqc/). Trimmomatic trimmed the adapters in the raw RNA-seq data. The quality controlled RNA-seq reads were mapped to reference human genome assembly hg19 using Bowtie (v0.12.7), with two mismatches and seed length 28. The expression abundance was measured by transcripts per million (TPM), which is independent of the mean expressed transcript length and is comparable across samples. Expression correlation was characterized by Pearson's correlation coefficient between TPM of all transcripts. Differentially expressed genes (DEGs) were identified through Cufflinks-Cuffdiff pipelines. The Cufflinks v2.1.1 was used to assemble transcripts and to estimate their abundance[55]. Cuffdiff v2.1.1 was then employed to identify DEGs[55]. Only genes adjusted p-value < 0.05) were considered as DEGs and used for further analysis. Clustering of genes was

implemented using the software program Cluster. Average linkage clustering with uncentered correlation was used. Software TreeView generated visual representations of clustering.Functional annotation tool DAVID was used for Gene Ontology (GO) enrichment.

6. Endothelial cell culture and assays

Endothelial cells were plated onto Collagen I-coated 6-well plates in EGM2 medium (Lonza). For immunofluorescence staining assay, endothelial cells were prepared on Collagen-coated chamber glass slides, fixed with 4% paraformaldehyde, permeabilized with 0.1% Triton X-100, then stained with primary antibody CD31 (1:200) overnight, subsequently stained with species-specific secondary antibodies conjugated to Alexa Fluor® 448 (1:400) .Nuclei were counterstained with DAPI (1:5000; Sigma). Then, the samples were examined under fluorescent microscope (Leica Microsystems). For capillary structure formation, growth factor–reduced Matrigel (BD Falcon) was added into a 96-well plate (60μL/well) and allowed to solidify for 30 minutes at 37°C. Cells suspended in EGM-2 were plated onto a gel matrix (40,000 cells/well) and incubated at 37°C for 12 hours.

7. Embryoid body random differentiation

Human ESCs were digested by Dispase (2 mg/ml, Invitrogen) for 5 min at confluence up to 80%, and then gently scraped by tip to form small aggregations. Then they were resuspended in F12 (Hyclone) supplemented with 20% Knockout Serum Replacement (KSR), 1% L-GlutaMax, 1% NEAA, 1% 2-Mecaptoenthanol (GIBICO). EBs were harvested and resuspended in fresh culture medium every three days until day 12 and lysed by Trizol for RNA preparation.

8. Myeloid differentiation on stromal cells

For myeloid cells generated in either OP9 or OP9-DL1 cell lines, $CD34^+$ HPC generated from H1 and H1-*GATA2*$^{-/-}$ in day 9 of coculture were added to OP9 or OP9-DL1 cells at a density of 50,000 per well of a 12-well-plate in MEM alpha supplemented with 10% FBS (HyClone Laboratories), MTG (100 Mm, 1-Thioglycerol, Sigma), IL3, IL6, GM-CSF, G-CSF and SCF (20ng/ml, PeproTech) for 12 days.

9. Real-time quantitative PCR (RT-qPCR)

Total RNA was extracted by Trizol (Invitrogen) and reverse-transcribed with RT-

PCR kit (TaKaRa). RT-qPCR was conducted with SYBR Green Premix EX TaqTM (TaKaRa) and a Thermal Cycler DiceTM Real Time System. Quantification of target genes was determined in comparison to the reference GAPDH gene, since its expression remains constant during ESC differentiation. All primers were shown in Table S1.

10. hESCs culture and differentiation in OP9 coculture

H1 ES cell line was obtained from Dr. Duanqing Pei's laboratory and at the passage of 38, gene targeting was performed. hESCs were cultured in mTeSR1 medium (Stem cell) with matrigel (BD) coated plate in incubator with 37°C, 5% CO_2 and 95% humidity. Cultures were passaged every three to four days by dissociation with EDTA.

For hESCs differentiation in OP9 coculture, OP9 cells (ATCC) were plated and cultured for 4 days to a overgrown status in MEM alpha (GIBICO) supplemented with 20% FBS (ExCell) and two wells (6-well-plate) of ES cells with 80% confluence were digested into small cell aggregates by Dispase (GIBICO, 2 mg/ml) association and followed with scrape. Then, the aggregates were added to OP9 cells in 10 ml MEM alpha supplemented with 10% FBS (HyClone Laboratories) and 100 μM MTG (1-Thioglycerol, Sigma). 20ml fresh medium was replaced on day 1, and replaced half with fresh medium every other day on day 4, 6, and 8. The hESCs/OP9 cocultures were incubated in condition with 37°C, 5% CO_2 and 95% humidity up to 9 days. For cell harvesting, cocultures were digested by 0.25% trypsin-0.5mM EDTA for 15 min at 37°C. After digestion, cells were filtered through a 70 μm cell strainer (BD Falcon) for further analysis.
